# Catalytic Oxidation of Chlorobenzene over HSiW/CeO_2_ as a Co-Benefit of NO*_x_* Reduction: Remarkable Inhibition of Chlorobenzene Oxidation by NH_3_

**DOI:** 10.3390/ma17040828

**Published:** 2024-02-08

**Authors:** Leyuan Dong, Keyu Jiang, Qi Shen, Lijuan Xie, Jian Mei, Shijian Yang

**Affiliations:** School of Environment & Ecology, Jiangnan University, Wuxi 214122, China

**Keywords:** chlorobenzene oxidation, HSiW/CeO_2_, the Mars–van–Krevelen mechanism, the Eley-Rideal mechanism, NH_3_ inhibition

## Abstract

There is an urgent need to develop novel and high-performance catalysts for chlorinated volatile organic compound oxidation as a co-benefit of NO*_x_*. In this work, HSiW/CeO_2_ was used for chlorobenzene (CB) oxidation as a co-benefit of NO*_x_* reduction and the inhibition mechanism of NH_3_ was explored. CB oxidation over HSiW/CeO_2_ primarily followed the Mars–van–Krevelen mechanism and the Eley-Rideal mechanism, and the CB oxidation rate was influenced by the concentrations of surface adsorbed CB, Ce^4+^ ions, lattice oxygen species, gaseous CB, and surface adsorbed oxygen species. NH_3_ not only strongly inhibited CB adsorption onto HSiW/CeO_2_, but also noticeably decreased the amount of lattice oxygen species; hence, NH_3_ had a detrimental effect on the Mars–van–Krevelen mechanism. Meanwhile, NH_3_ caused a decrease in the amount of oxygen species adsorbed on HSiW/CeO_2_, which hindered the Eley-Rideal mechanism of CB oxidation. Hence, NH_3_ significantly hindered CB oxidation over HSiW/CeO_2_. This suggests that the removal of NO*_x_* and CB over this catalyst operated more like a two-stage process rather than a synergistic one. Therefore, to achieve simultaneous NO*_x_* and CB removal, it would be more meaningful to focus on improving the performances of HSiW/CeO_2_ for NO*_x_* reduction and CB oxidation separately.

## 1. Introduction

Volatile organic compounds (VOCs) are important precursors for the formation of secondary pollutants such as fine particles and ozone, which leads to atmospheric environmental problems such as haze and photochemical smog [1,2,3]. Among them, chlorinated volatile organic compounds (Cl-VOCs) are particularly concerning due to their high toxicity towards living organisms and their persistence in the environment [4,5]. Therefore, there is a pressing need to strictly regulate and control the release of these pollutants. In various industrial processes, such as steel sintering, waste incineration, and coking, Cl-VOCs are often found in coexistence with nitrogen oxides (NO*_x_*, *x* = 1 and 2) in flue gases [6]. Currently, selective catalytic reduction (SCR) of NO*_x_* with NH_3_ has been considered a promising technology to control NO*_x_* emissions from these industrial processes [7]. Therefore, the catalytic oxidation of harmful Cl-VOCs to non-toxic CO_2_, H_2_O, and soluble HCl using SCR catalysts may be an economically viable and environmentally friendly technology to control the emission of Cl-VOCs from flue gases in these industries [8].

However, the traditional commercial SCR catalyst (i.e., V_2_O_5_-WO_3_/TiO_2_) has encountered some challenges when it comes to the catalytic oxidation of Cl-VOCs, which include the low activity and accumulation of polychlorinated species [9]. In an effort to overcome these limitations, some researchers have attempted to enhance the performance of V_2_O_5_-WO_3_/TiO_2_ for the catalytic oxidation of Cl-VOCs through various modifications. For instance, Li et al. discovered that the loading of Ru significantly reduced the kinetic barriers associated with both C–Cl cleavage and HCl formation on V_2_O_5_-WO_3_/TiO_2_, resulting in a substantial improvement in the effectiveness of chlorobenzene (CB) oxidation [10]. Similarly, Si et al. observed that loading Sb onto V_2_O_5_-WO_3_/TiO_2_ not only weakened the Lewis acid sites on the surface but also enhanced the formation of oxygen vacancies. Therefore, the Sb-doped V_2_O_5_-WO_3_/TiO_2_ catalyst had high CB conversion efficiency while minimizing the formation of polychlorinated species [11]. However, despite these modifications, the CB oxidation activity and selectivities of CO_2_ and HCl of these modified V_2_O_5_-WO_3_/TiO_2_ catalysts still fall short of being satisfactory. Additionally, these modified catalysts exhibit low N_2_ selectivity, have a narrow temperature range for optimal performance, and rely on the use of toxic vanadium pentoxide. These drawbacks restrict the application of these modified V_2_O_5_-WO_3_/TiO_2_ catalysts for NO*_x_* reduction. As a result, there is a significant need to develop novel and highly efficient catalysts specifically for the catalytic oxidation of Cl-VOCs while also providing the co-benefit of NO*_x_* reduction.

The catalyst used to remove NO*_x_* and Cl-VOCs should possess excellent surface acidity and redox properties. Ce-based oxides generally have prominent oxygen mobility, high oxygen storage and release capacity, and even some acidic properties [12], which have been widely utilized in the reduction of NO*_x_* and the catalytic oxidation of Cl-VOCs. Further research conducted by Zhang et al. revealed that Ce-Ti amorphous oxide demonstrated remarkable SCR activity across a wide temperature range. This was attributed to the presence of abundant active sites provided by the Ce–O–Ti species [13]. Another study by Peng et al. reported that CeO_2_-WO_3_ exhibited exceptional SCR activity and displayed resistance against alkali metal poisoning [14]. Jia et al. observed that S-Ce_0.7_Zr_0.3_O_2_ showed superior CB oxidation activity and lower by-product selectivity. This was explained by the synergistic effect of Lewis and Brønsted acid sites present on the catalyst [15]. Additionally, Weng et al. discovered that sulfide-modified NiO/CeO_2_ displayed excellent CB oxidation activity and selectivity towards CO*_x_*. This was attributed to the enhanced Lewis acidity and the presence of surface oxygen vacancies [16]. Consequently, based on these findings, Ce-based oxides may be promising alternatives to the commercial V_2_O_5_-WO_3_/TiO_2_ catalyst for the catalytic oxidation of Cl-VOCs. This substitution is advantageous, as it provides the additional co-benefit of NO*_x_* reduction.

Previous studies have found that CeO_2_ modified by silicotungstic acid (HSiW/CeO_2_) not only exhibited excellent SCR performance [17] but also displayed remarkable efficacy in catalyzing the oxidation of CB [18]. This dual functionality of HSiW/CeO_2_ for NO*_x_* reduction and CB oxidation makes it a promising candidate for the simultaneous removal of NO*_x_* and Cl-VOCs. In this work, the performance of HSiW/CeO_2_ for CB (the model compound for Cl-VOCs) oxidation as a co-benefit of NO*_x_* reduction was investigated, and the inhibition mechanism of NH_3_ on CB oxidation over HSiW/CeO_2_ was deeply explored. The results from in situ DRIFTS and kinetics studies revealed that CB oxidation over HSiW/CeO_2_ mainly followed the Mars–van–Krevelen mechanism and the Eley–Rideal mechanism, and the rate of CB oxidation was primarily influenced by the concentrations of surface adsorbed CB, Ce^4+^ ions, lattice oxygen species, gaseous CB, and surface adsorbed oxygen species. NH_3_ was found to inhibit the adsorption of CB onto HSiW/CeO_2_ and decrease the amount of lattice oxygen species, thereby significantly suppressing the contribution of the Mars–van–Krevelen mechanism to CB oxidation. Additionally, NH_3_ reduced the amount of oxygen species adsorbed on HSiW/CeO_2_, leading to a remarkable inhibition of the Eley–Rideal mechanism. Consequently, NH_3_ greatly inhibited the catalytic oxidation of CB over HSiW/CeO_2_, resulting in a close to two-stage removal of NO*_x_* and CB rather than a synergistic removal.

## 2. Experimental Section

### 2.1. Catalyst Preparation

CeO_2_ was obtained from the calcination of Ce(NO_3_)_3_·6H_2_O at 300 °C for 120 min in air. The resulting CeO_2_ weighing 20 g was then immersed in a solution of HSiW with a concentration of 20 g L^−1^ and a volume of 500 mL for 120 min under the condition of an ice bath. After the immersion, the mixture was subjected to centrifugation, followed by drying. Subsequently, the CeO_2_ was calcined at 500 °C for 180 min in air to obtain HSiW/CeO_2_. In addition, V_2_O_5_-WO_3_/TiO_2_, containing 1% of V_2_O_5_ and 10% of WO_3_, was prepared by the traditional impregnation method.

### 2.2. Catalytic Performance Evaluation

The catalytic performances of NO*_x_* reduction and CB oxidation were evaluated in a fixed-bed quartz reactor at temperatures ranging from 250 to 450 °C. The catalyst mass was typically 30 mg, and the total flow rate of the gas was 200 mL min^−1^. This resulted in a gas hourly space velocity (GHSV) of 400,000 cm^3^ g^−1^ h^−1^. The simulated flue gas generally contained 500 ppm NO*_x_* (during use), 500 ppm NH_3_ (during use), 100 ppm CB (during use), 5% O_2_, 100 ppm SO_2_ (during use), 8% H_2_O (during use), and N_2_ balance. The concentrations of NO, NO_2_, N_2_O, NH_3_, CB, CO, CO_2_, and HCl in the outlet of the reactor were measured online using an infrared gas analyzer (Thermo Fisher, IGS Analyzer, Waltham, MA, USA). The catalytic efficiency was evaluated based on the following parameters: NO*_x_* conversion efficiency, N_2_ selectivity, CB conversion efficiency, and CO*_x_* selectivity (which includes both CO and CO_2_). These parameters were calculated using specific equations:

(1) $${\mathrm{NO}}_{x}\;\mathrm{conversion}=\frac{{\left[{\mathrm{NO}}_{x}\right]}_{\mathrm{in}}-{\left[{\mathrm{NO}}_{x}\right]}_{\mathrm{out}}}{{\left[{\mathrm{NO}}_{x}\right]}_{\mathrm{in}}}\times 100\%$$

(2) $$N_{2}\;\mathrm{selectivity}=\left(1-\frac{2{\left[N_{2}O\right]}_{\mathrm{out}}}{{\left[{\mathrm{NO}}_{x}\right]}_{\mathrm{in}}-{\left[{\mathrm{NO}}_{x}\right]}_{\mathrm{out}}+{\left[{\mathrm{NH}}_{3}\right]}_{\mathrm{in}}-{\left[{\mathrm{NH}}_{3}\right]}_{\mathrm{out}}}\right)\times 100\%$$

(3) $$\mathrm{CB}\;\mathrm{conversion}=\frac{{\left[\mathrm{CB}\right]}_{\mathrm{in}}-{\left[\mathrm{CB}\right]}_{\mathrm{out}}}{{\left[\mathrm{CB}\right]}_{\mathrm{in}}}\times 100\%$$

(4) $${\mathrm{CO}}_{x}\;\mathrm{selectivity}=\frac{{\left[{\mathrm{CO}}_{x}\right]}_{\mathrm{out}}}{6({\left[\mathrm{CB}\right]}_{\mathrm{in}}-{\left[\mathrm{CB}\right]}_{\mathrm{out}})}\times 100\%$$

where [NO*_x_*]_in_, [NH_3_]_in_, and [CB]_in_ are the concentrations of NO*_x_*, NH_3_, and CB in the inlet, respectively, and [NO*_x_*]_out_, [NH_3_]_out_, [CB]_out_, and [CO*_x_*]_out_ are the concentrations of NO*_x_*, NH_3_, CB, and CO*_x_* in the outlet, respectively.

### 2.3. Catalyst Characterization

X-ray diffraction pattern (XRD), BET surface area, X-ray photoelectron spectra (XPS), and X-ray fluorescence (XRF) were measured by an X-ray diffractometer (Bruker-AXS D8 ADVANCE, Billerica, MA, USA), N_2_ adsorption analyzer (Quantachrome 2200e, Boynton Beach, FL, USA), X-ray photoelectron spectroscope (Thermo Fisher ESCALAB 250, Waltham, MA, USA), and X-ray fluorescence analyzer (XRF, Thermo Fisher ARL, Waltham, MA, USA), respectively.

Temperature-programmed desorption of CB (CB-TPD) was conducted on the same fixed-bed quartz reactor that was used for the catalytic performance evaluation. 200 mg of HSiW/CeO_2_ was firstly pretreated with 200 mL min^−1^ of 5% O_2_/N_2_ at 400 °C for 60 min and then cooled to 50 °C. Afterward, HSiW/CeO_2_ was exposed to 100 ppm CB and 500 ppm NH_3_ (during use) for 60 min. Finally, HSiW/CeO_2_ was purged by 100 mL min^−1^ of N_2_ from 50 to 600 °C at the heating rate of 10 °C min^−1^.

In situ diffuse reflectance infrared Fourier transform spectroscopy (DRIFTS) was conducted on a Fourier transform infrared spectrometer (Thermo Fisher, Nicolet iS50, Waltham, MA, USA) equipped with an MCT detector. The spectra were collected at a resolution of 4 cm^−1^ and over 32 scans.

## 3. Results and Discussion

### 3.1. Performances for NO_x_ Reduction and CB Oxidation

#### 3.1.1. Activity and Product Selectivity

**Figure 1:**
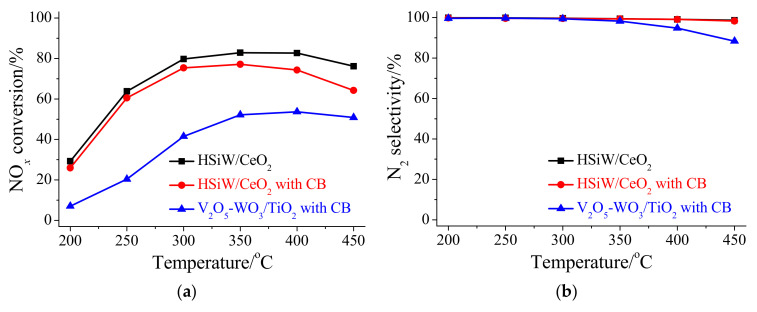

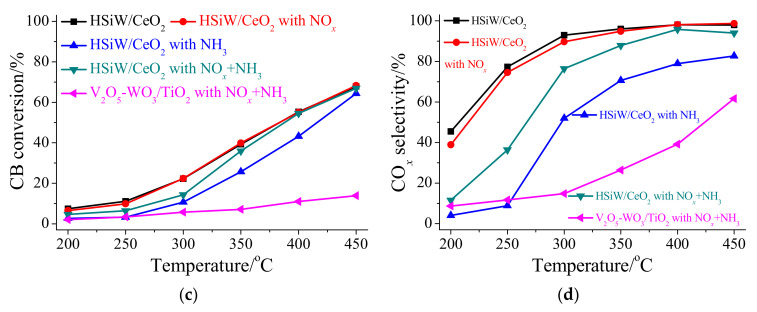
(**a**) NO*_x_* conversion efficiency, (**b**) N_2_ selectivity, (**c**) CB conversion efficiency, and (**d**) CO*_x_* selectivity of HSiW/CeO_2_ and V_2_O_5_-WO_3_/TiO_2_. Reaction conditions: [NO*_x_*] = 500 ppm (during use), [NH_3_] = 500 ppm (during use), [CB] = 100 ppm (during use), [O_2_] = 5%, catalyst mass = 30 mg, total flow rate = 200 mL min^−1^, and GHSV = 400,000 cm^3^ g^−1^ h^−1^.

HSiW/CeO_2_ exhibited excellent NO*_x_* reduction activity in a broad temperature range under a high GHSV of 400,000 cm^3^ g^−1^ h^−1^, and the NO*_x_* conversion efficiency was higher than 60% at 250–450 °C (Figure 1a). Meanwhile, little N_2_O was formed during NO*_x_* reduction over HSiW/CeO_2_ at 200–450 °C (Figure S1), resulting in excellent N_2_ selectivity of approximately 100% (Figure 1b). Although NO*_x_* reduction over HSiW/CeO_2_ was slightly inhibited by CB, the NO*_x_* conversion still reached at least 60% at 250–450 °C in the presence of CB (Figure 1a). Meanwhile, the N_2_ selectivity of HSiW/CeO_2_ for NO*_x_* reduction scarcely changed when CB was present (Figure 1b). These results suggest that HSiW/CeO_2_ still had excellent performance for NO*_x_* reduction even in the presence of CB, which was also much better than that of V_2_O_5_-WO_3_/TiO_2_ (Figure 1a,b).

HSiW/CeO_2_ also showed excellent CB oxidation activity under a high GHSV of 400,000 cm^3^ g^−1^ h^−1^, and the CB conversion efficiency was approximately 23–68% at 300–450 °C (Figure 1c). Meanwhile, the catalytic oxidation of CB over HSiW/CeO_2_ showed excellent selectivity of CO*_x_*, and it reached higher than 95% at 300–450 °C (Figure 1b). This suggests that a low selectivity of other organic intermediates or by-products occurred during CB oxidation over HSiW/CeO_2_. After the introduction of NO*_x_*, neither the CB conversion efficiency nor CO*_x_* selectivity changed (Figure 1c,d). This suggests that the catalytic oxidation of CB over HSiW/CeO_2_ was barely affected by NO*_x_*. However, both the CB conversion efficiency and CO*_x_* selectivity of HSiW/CeO_2_ remarkably decreased after the introduction of NH_3_ (Figure 1c,d), suggesting that the catalytic oxidation of CB over HSiW/CeO_2_ was remarkably inhibited by NH_3_. Furthermore, the catalytic oxidation of CB over HSiW/CeO_2_ was also restrained by the coexistence of NO*_x_* and NH_3_, but the inhibition effect was not as obvious as that of NH_3_ alone (Figure 1c,d). Moreover, the performance of HSiW/CeO_2_ for CB oxidation in the presence of NO*_x_* and NH_3_ was also much better than that of V_2_O_5_-WO_3_/TiO_2_ (Figure 1a,b).

#### 3.1.2. Long-Term Stability

**Figure 2:**
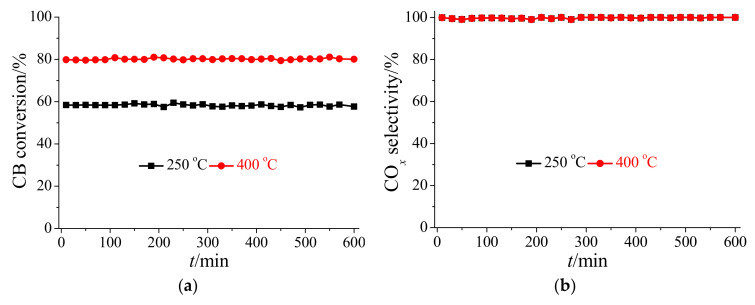
(**a**) CB conversion efficiency and (**b**) CO*_x_* selectivity of HSiW/CeO_2_ at 250 and 400 °C for a long time. Reaction conditions: [CB] = 100 ppm (during use), [O_2_] = 5%, catalyst mass = 50 mg, total flow rate = 200 mL min^−1^, and GHSV = 240,000 cm^3^ g^−1^ h^−1^.

Ce-based oxide catalysts are generally easily deactivated by Cl poisoning during CB oxidation [19,20], and thus the long-term stabilities of HSiW/CeO_2_ for CB oxidation were investigated at different reaction temperatures. Figure 2a shows that the CB conversion efficiency of HSiW/CeO_2_ was stable at approximately 58% at 250 °C for 600 min. Meanwhile, the CO*_x_* selectivity of HSiW/CeO_2_ stabilized at approximately 100% at 250 °C for 600 min (Figure 2b). These results suggest that HSiW/CeO_2_ showed excellent stability for CB oxidation at low reaction temperatures. As the reaction temperature increased to 400 °C, both the CB conversion efficiency and CO*_x_* selectivity of HSiW/CeO_2_ were stable at approximately 80% and 100% for 600 min, respectively (Figure 2). This suggests that HSiW/CeO_2_ exhibited excellent stability for CB oxidation at high reaction temperatures. Therefore, HSiW/CeO_2_ showed excellent resistance to Cl poisoning during CB oxidation, resulting in excellent stability of CB conversion efficiency and CO*_x_* selectivity.

### 3.2. Characterization

#### 3.2.1. XRD and BET Surface Area

The XRD pattern of HSiW/CeO_2_ showed a remarkable similarity to that of cerianite (CeO_2_), which was characterized by a specific diffraction pattern known as JPCDS 43-1002. This suggests that the grafting of HSiW onto CeO_2_ did not significantly alter the cubic fluorite structure of CeO_2_. Additionally, the BET surface area of HSiW/CeO_2_ was approximately 54.4 m^2^ g^−1^.

#### 3.2.2. XPS

**Figure 3:**
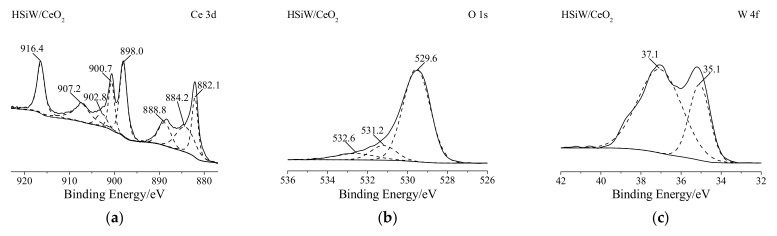

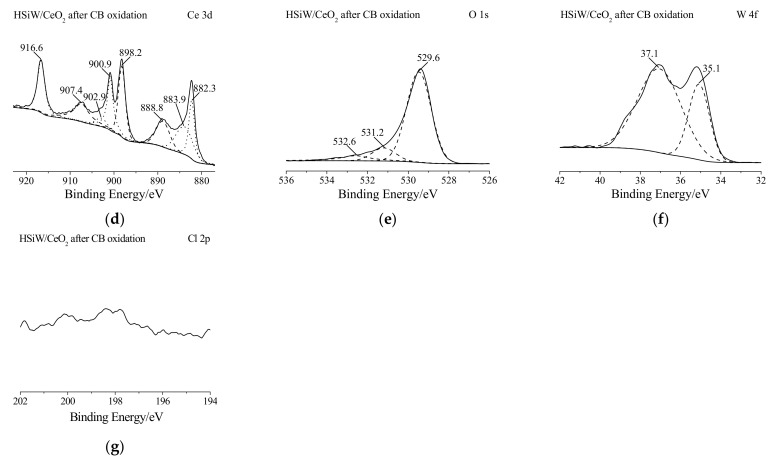
XPS spectra of HSiW/CeO_2_ and HSiW/CeO_2_ after CB oxidation in the spectral regions of (**a**,**d**) Ce 3d, (**b**,**e**) O 1s, (**c**,**f**) W 4f, and (**g**) Cl 2p.

The Ce 3d binding energies for HSiW/CeO_2_ were observed at 882.1, 885.4, 888.8, 898.0, 900.7, 902.8, 907.2, and 916.4 eV (Figure 3a), which were attributed to two different oxidation states of Ce: Ce^3+^ (including 885.4 and 902.8 eV) and Ce^4+^ (including 882.1, 888.8, 898.0, 900.7, 907.2, and 916.4 eV) [21,22]. Similarly, the O 1s binding energies for HSiW/CeO_2_ were observed at 529.6, 531.2, and 532.6 eV (Figure 3b). The binding energy at 529.6 eV corresponded to the lattice oxygen, while the binding energies at 531.2 and 532.6 eV were related to the adsorbed oxygen and oxygen in the HSiW, respectively [17,22]. In addition, the W 4f binding energies for HSiW/CeO_2_ were observed at 35.1 and 37.1 eV (Figure 3c), which were attributed to W 4f_7/2_ and W 4f_5/2_ of W^6+^ in the HSiW, respectively [23]. These results suggest that both Ce^3+^ and Ce^4+^ species, as well as lattice oxygen and adsorbed oxygen species, were present on HSiW/CeO_2_. Meanwhile, the Keggin structure of HSiW remained intact in HSiW/CeO_2_. Furthermore, the percentages of W and Ce species in HSiW/CeO_2_ resulted from XPS analysis, and the contents of W and Ce species in HSiW/CeO_2_ resulted from XRF analysis, as compared in Table S1. Table S1 revealed that the percentage of W species on the surface of HSiW/CeO_2_ was significantly larger than its content within HSiW/CeO_2_, suggesting that HSiW was predominantly present on the surface of CeO_2_.

After conducting CB oxidation for 600 min, the Ce 3d, W 4f, and O 1s spectra of HSiW/CeO_2_ did not vary significantly (Figure 3d–f). Additionally, there was no detectable peak corresponding to Cl 2p in the spectrum of HSiW/CeO_2_ after the 600 min CB oxidation (Figure 3g). These findings indicate that there was very little deposition of Cl species on the surface of HSiW/CeO_2_ during the CB oxidation process. Hence, HSiW/CeO_2_ displayed remarkable resilience against Cl poisoning, which has been illustrated in Figure 2.

### 3.3. Mechanism of CB Oxidation

The potential mechanism of CB oxidation over HSiW/CeO_2_ generally followed the Mars–van–Krevelen mechanism (i.e., gaseous CB was firstly physically adsorbed on the catalyst, which was then oxidized by the lattice oxygen species to form the final product, and finally gaseous O_2_ replenished the lattice oxygen species consumed) and the Eley–Rideal mechanism (i.e., gaseous CB reacted with the surface adsorbed oxygen species to form the final product) [24,25].

The catalytic oxidation of CB over HSiW/CeO_2_ through the Mars–van–Krevelen mechanism can be approximately expressed as:

(5) $$C_{6}H_{5}{\mathrm{Cl}}_{\left(g\right)}\to C_{6}H_{5}{\mathrm{Cl}}_{\left(\mathrm{ad}\right)}$$

(6) $$C_{6}H_{5}{\mathrm{Cl}}_{\left(\mathrm{ad}\right)}+28\equiv {\mathrm{Ce}}^{4+}+14O^{2-}\to 6{\mathrm{CO}}_{2}+\mathrm{HCl}+2H_{2}O+28\equiv {\mathrm{Ce}}^{3+}$$

(7) $$\equiv {\mathrm{Ce}}^{3+}+O_{2(g)}\to \equiv {\mathrm{Ce}}^{4+}+2O^{2-}$$

The catalytic oxidation of CB over HSiW/CeO_2_ through the Eley–Rideal mechanism can approximately be expressed as:

(8) $$O_{2(g)}\to 2O_{\left(\mathrm{ad}\right)}$$

(9) $$C_{6}H_{5}{\mathrm{Cl}}_{\left(g\right)}+14O_{\left(\mathrm{ad}\right)}\to 6{\mathrm{CO}}_{2}+\mathrm{HCl}+2H_{2}O$$

**Figure 4:**
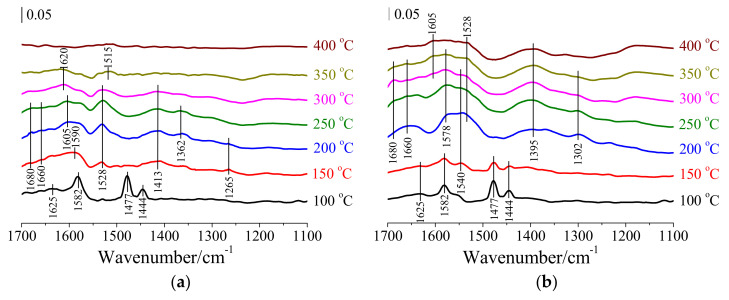
(**a**) In situ DRIFTS spectra of passing O_2_ over HSiW/CeO_2_ pre-adsorbed by CB from 100 to 400 °C. (**b**) In situ DRIFTS spectra of passing CB+O_2_ over HSiW/CeO_2_ from 100 to 400 °C.

In order to examine the role of the Mars–van–Krevelen mechanism in the catalytic oxidation of CB over HSiW/CeO_2_, in situ DRIFTS of passing O_2_ over HSiW/CeO_2_ pre-adsorbed by CB at temperatures ranging from 100 to 400 °C were conducted. Upon exposure to CB at 100 °C for 30 min, four distinctive bands were observed at 1444, 1477, 1582, and 1625 cm^−1^ (Figure 4a). The bands at 1444 and 1477 cm^−1^ were attributed to the stretching vibration of the C=C bond in CB adsorbed on Brønsted acid sites due to Ce–OH in CeO_2_, and the band at 1477 cm^−1^ was also ascribed to the stretching vibration of the C=C bond in CB adsorbed on Brønsted acid sites due to W–OH in HSiW. The band at 1582 cm^−1^ was assigned to the stretching vibration of the C=C bond in CB adsorbed on Lewis acid sites, which were formed by Ce^3+^/Ce^4+^ species in CeO_2_. The band at 1625 cm^−1^ corresponded to the out-plane bending vibration of the C–H bond in the aromatic ring of CB adsorbed on Lewis acid sites, which were associated with W^6+^ species in HSiW. These observations indicated the adsorption of CB on the surface of HSiW/CeO_2_. When the reaction temperature was raised to 150 °C, the bands corresponding to the adsorbed CB almost disappeared, while six new bands appeared at 1265, 1413, 1528, 1590, 1660, and 1680 cm^−1^ (Figure 4a). The bands at 1265 and 1590 cm^−1^ were attributed to the stretching vibrations of the C–O bond in phenolate species and the C=C bond in the aromatic ring, respectively [26]. This suggests that the phenolate species were formed through the cleavage of the C–Cl bond in CB via a nucleophilic substitution reaction with lattice oxygen species. The bands at 1660 and 1680 cm^−1^ were assigned to the stretching vibration of the C=O bond in *p*-benzoquinone and o-benzoquinone species, respectively [27], indicating that some phenolate species were attacked by lattice oxygen species, resulting in the formation of benzoquinone. The band at 1528 cm^−1^ was associated with the symmetric stretching vibration of COO^−^ groups, indicating the presence of maleic anhydride species [28]. This suggests that certain benzoquinone species were attacked by lattice oxygen, leading to the cleavage of the aromatic ring and the formation of maleic anhydride. The band at 1413 cm^−1^ corresponded to the asymmetric stretching vibration of COO^−^ groups [29], suggesting that the maleic anhydride species were further oxidized to form acetate species. With further increase in the reaction temperature to 200 °C, two additional bands at 1362 and 1605 cm^−1^ were observed, which were attributed to the stretching vibration of COO^−^ groups from acetate species [30]. This indicates that the remaining maleic anhydride species were being further oxidized. As the reaction temperature reached 300 °C, the bands corresponding to phenolate species, benzoquinone species, and maleic anhydride species nearly disappeared. Only two new bands at 1515 and 1620 cm^−1^ were present, which were attributed to the stretching vibrations of COO^−^ groups from acetate species and –OH groups from water molecules, respectively [31]. This indicates that some acetate species were undergoing further oxidation to form the final products. These results strongly suggest that CB adsorbed on HSiW/CeO_2_ can be oxidized by lattice oxygen species, ultimately leading to the formation of the final products. Hence, the Mars–van–Krevelen mechanism played a significant role in the catalytic oxidation of CB over HSiW/CeO_2_.

According to Reaction (6), the rate of HSiW/CeO_2_ for CB oxidation through the Mars–van–Krevelen mechanism (i.e., *δ*_MvK_) can be approximately expressed as:

(10) $$\delta_{\mathrm{MvK}}=-\frac{d[C_{6}H_{5}{\mathrm{Cl}}_{\left(g\right)}]}{\mathrm{dt}}=k_{1}{[C}_{6}H_{5}{\mathrm{Cl}}_{\left(\mathrm{ad}\right)}]{\left[\equiv {\mathrm{Ce}}^{4+}\right]}^{\alpha }{\left[O^{2-}\right]}^{\beta }$$

where *k*_1_, [C_6_H_5_Cl_(ad)_], [≡Ce^4+^], [O^2−^], *α*, and *β* are the kinetic constant of Reaction (6), amounts of CB adsorbed, Ce^4+^ ions and lattice oxygen species on the surface, and reaction orders of Reaction (6) with respect to the amounts of surface Ce^4+^ and O^2−^, respectively.

According to Reaction (9), the rate of HSiW/CeO_2_ for CB oxidation through the Eley–Rideal mechanism (i.e., *δ*_E-R_) can be approximately expressed as:

(11) $$\delta_{E-R}=-\frac{d[C_{6}H_{5}{\mathrm{Cl}}_{\left(g\right)}]}{\mathrm{dt}}=k_{2}{[C}_{6}H_{5}{\mathrm{Cl}}_{\left(g\right)}]{\left[O_{\left(\mathrm{ad}\right)}\right]}^{\gamma }$$

where *k*_2_, [C_6_H_5_Cl_(g)_], [O_(ad)_], and *γ* are the kinetic constant of Reaction (9), amounts of gaseous CB in the flue gas and surface adsorbed oxygen species, and reaction order of Reaction (9) with respect to the amount of surface adsorbed oxygen species, respectively.

Therefore, the rate of HSiW/CeO_2_ for CB oxidation can be approximately expressed as:

(12) $$\begin{array}{l} \delta =\delta_{\mathrm{MvK}}+\delta_{E-R}\\ =k_{1}{[C}_{6}H_{5}{\mathrm{Cl}}_{\left(\mathrm{ad}\right)}]{\left[\equiv {\mathrm{Ce}}^{4+}\right]}^{\alpha }{\left[O^{2-}\right]}^{\beta }+k_{2}{[C}_{6}H_{5}{\mathrm{Cl}}_{\left(g\right)}]{\left[O_{\left(\mathrm{ad}\right)}\right]}^{\gamma } \end{array}$$

The concentration of CB in the flue gas was found to be generally high, with an approximate concentration of 100 ppm. This suggests that HSiW/CeO_2_ was nearly saturated with the adsorption of CB. Therefore, the amount of CB adsorbed on the surface of HSiW/CeO_2_ can be considered as a constant. Furthermore, both Ce^4+^ and O^2−^ can be quickly recovered through Reaction (7), so the concentrations of Ce^4+^ and O^2−^ ions on HSiW/CeO_2_ can also be regarded as constants. In addition, the concentration of O_2_ in the flue gas was approximately 5%, which was approximately 500 times that of CB. This suggests that the decrease in the concentration of O_(ad)_ on HSiW/CeO_2_ due to CB oxidation (i.e., Reaction (9)) can be approximately neglected. Therefore, the concentration of O_(ad)_ on HSiW/CeO_2_ can be deemed as a constant. As suggested by Equation (11), it was anticipated that the rate of CB oxidation would exhibit an excellent linear relationship with the CB concentration. The intercept and slope of this relationship can be used to describe the kinetic constants of CB oxidation through the Mars–van–Krevelen mechanism (i.e., *k*_MvK_) and the Eley–Rideal mechanism (i.e., *k*_E-R_), respectively.

Therefore, Equation (12) can be approximately revised as:

(13) $$\delta =\delta_{\mathrm{MvK}}+\delta_{E-R}=k_{\mathrm{MvK}}+k_{E-R}{[C}_{6}H_{5}{\mathrm{Cl}}_{\left(g\right)}]$$

**Figure 5:**
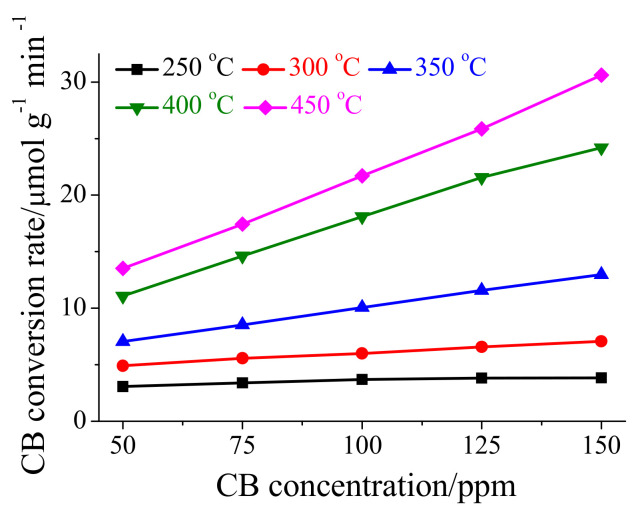
Dependence of CB conversion rate on the CB concentration. Reaction conditions: [CB] = 50–150 ppm, [O_2_] = 5%, catalyst mass = 3–30 mg, total flow rate = 200 mL min^−1^, and GHSV = 400,000–4,000,000 cm^3^ g^−1^ h^−1^.

In order to determine the kinetic constants of CB oxidation using the Mars–van–Krevelen mechanism and the Eley–Rideal mechanism, the kinetics experiment of CB oxidation over HSiW/CeO_2_ at 250–450 °C with lower than 15% of CB conversion efficiency was performed, and the dependence of CB conversion rate on CB concentration was shown in Figure 5. Figure 5 shows that the CB conversion rate of HSiW/CeO_2_ significantly increased as the CB concentration increased. Furthermore, the relationship between the CB oxidation rate and the CB concentration was found to be linear, indicating a direct dependence. This result was in agreement with the assumption stated in Equation (13). To further analyze the data, a linear regression analysis was performed on Figure 5, using Equation (13) as the basis. The obtained slope, intercept, and regression coefficient of the linear regression analysis are all listed in Table 1.

According to the data shown in Table 1, the values of the intercept (*k*_MvK_) were found to be approximately 2.77, 3.89, 4.07, 4.63, and 4.79 µmol g^−1^ min^−1^ at 250, 300, 350, 400, and 450 °C, respectively. Similarly, Table 1 also shows that the values of the slope (*k*_E-R_) were approximately 0.008, 0.021, 0.060, 0.132, and 0.170 µmol g^−1^ min^−1^ at 250, 300, 350, 400, and 450 °C, respectively. Based on these, it can be inferred that the catalytic oxidation of CB over HSiW/CeO_2_ was influenced not only by the Mars–van–Krevelen mechanism but also by the Eley–Rideal mechanism. Additionally, it was revealed that the rate of CB oxidation over HSiW/CeO_2_ via the Eley–Rideal mechanism was directly proportional to the CB concentration, as indicated by Equation (11). Therefore, when the CB concentration was approximately 346, 185, 68, 49, and 28 ppm at 250, 300, 350, 400, and 450 °C, respectively, the CB oxidation rate through the Eley–Rideal mechanism was equal to that through the Mars–van–Krevelen mechanism. This suggests that at these specific CB concentrations, both mechanisms contribute equally to CB oxidation over HSiW/CeO_2_. However, when the CB concentration was lower than the aforementioned values at each temperature, the CB oxidation rate through the Mars–van–Krevelen mechanism was larger than that through the Eley–Rideal mechanism. This indicates that at lower CB concentrations, the Mars–van–Krevelen mechanism played a more dominant role in CB oxidation over HSiW/CeO_2_. On the other hand, when the CB concentration was higher than the stated values, the CB oxidation rate through the Eley–Rideal mechanism was larger than that through the Mars–van–Krevelen mechanism. This implies that at higher CB concentrations, the Eley–Rideal mechanism became more significant in CB oxidation over HSiW/CeO_2_. Considering that the CB concentration in the flue gas was generally approximately 100 ppm, the CB oxidation rate of HSiW/CeO_2_ through the Mars–van–Krevelen mechanism was larger than that through the Eley–Rideal mechanism at 250–300 °C, but smaller than that through the Eley–Rideal mechanism at 350–450 °C. Therefore, the catalytic oxidation of CB over HSiW/CeO_2_ was influenced by both the temperature and the CB concentration. The Mars–van–Krevelen mechanism appeared to be more important at lower temperatures and lower CB concentrations, while the Eley–Rideal mechanism became more dominant at higher temperatures and higher CB concentrations.

Although the adsorption of CB onto HSiW/CeO_2_ (i.e., Reaction (5)) was hindered by the increase in reaction temperature increased, both Reactions (6) and (7) were greatly accelerated. Hence, the value of *k*_MvK_ was observed to gradually increase with the rise in reaction temperature (Table 1). On the other hand, the adsorption of O_2_ onto HSiW/CeO_2_ (i.e., Reaction (8)) was suppressed with the increase in reaction temperature, while Reaction (9) was significantly accelerated. Thus, the *k*_E-R_ value also displayed a gradual increase with the elevation of reaction temperature (Table 1). Therefore, the catalytic oxidation of CB over HSiW/CeO_2_ was noticeably facilitated with the increase in reaction temperature, as illustrated in Figure 1c.

To gain a deeper understanding of the reaction pathway of CB oxidation over HSiW/CeO_2_ through the Eley–Rideal mechanism, in situ DRIFTS of passing CB+O_2_ over HSiW/CeO_2_ was performed at different temperatures ranging from 100 to 400 °C. In addition to the bands corresponding to adsorbed CB at 1444, 1477, 1582, and 1625 cm^−1^, benzoquinone species at1660 and 1680 cm^−1^, maleic anhydride species at 1528 cm^−1^, and acetate species at 1605 cm^−1^, four new bands at 1302, 1395, 1540, and 1578 cm^−1^ were observed on HSiW/CeO_2_ (Figure 4b). The bands at 1302, 1540, and 1578 cm^−1^ were assigned to the stretching vibrations of the C–O bond in phenolate species (1302 cm^−1^) and the C=C bond in the aromatic ring (1540 and 1578 cm^−1^), respectively [32]. Meanwhile, the band at 1395 cm^−1^ was attributed to the stretching vibration of the –CH_2_– bond in acetate species [30]. Their results suggest that the intermediates of CB oxidation over HSiW/CeO_2_ through the Eley–Rideal mechanism at most included the phenolate species, benzoquinone species, maleic anhydride species, and acetate species. Therefore, the reaction pathway of CB oxidation over HSiW/CeO_2_ through the Eley–Rideal mechanism may be the same as or simpler than that observed in the Mars–van–Krevelen mechanism.

**Figure 6:**
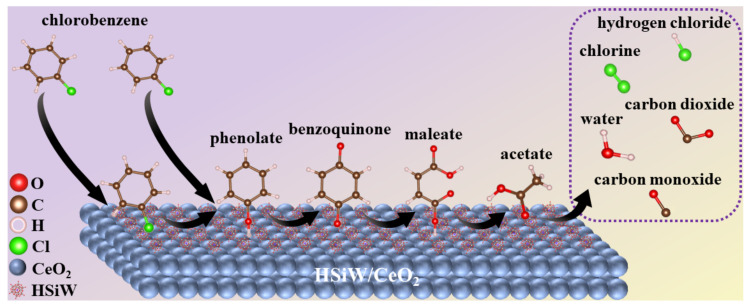
A credible reaction pathway of CB oxidation over HSiW/CeO_2_.

Based on the analysis results of in situ DRIFTS and reaction kinetics, it was found that the catalytic oxidation of CB over HSiW/CeO_2_ primarily followed two mechanisms: the Mars–van–Krevelen mechanism and the Eley–Rideal mechanism. Therefore, a credible reaction pathway of CB oxidation over HSiW/CeO_2_ was summarized in Figure 6: (1) A small portion of gaseous CB molecules were adsorbed onto the Brønsted acid sites of HSiW and CeO_2_ in HSiW/CeO_2_, as well as the Lewis acid sites of CeO_2_ in HSiW/CeO_2_. (2) The C–Cl bond in a fraction of the absorbed CB molecules, as well as most of the gaseous CB molecules, underwent a cleavage reaction through nucleophilic substitution with the lattice oxygen species. This reaction led to the formation of phenolate species. (3) The phenolate species were then attacked by the lattice oxygen species through an electrophilic substitution reaction, resulting in the formation of benzoquinone species. (4) The aromatic ring in the benzoquinone species was cleaved through the attack of the lattice oxygen species, leading to the formation of maleic anhydride species. (5) The maleic anhydride species underwent further deep oxidation, first transforming into acetate species and ultimately oxidizing into CO_2_, CO, and H_2_O. Moreover, the Cl species present in HSiW/CeO_2_ were rapidly eliminated. This was achieved through two potential reactions: the dissociatively adsorbed Cl reacting with surface hydroxyl groups to form HCl, or the Cl species reacting with other compounds to produce Cl_2_, which is known as the Deacon reaction [10].

### 3.4. Inhibition Mechanism of NH_3_ on CB Oxidation

Equation (12) indicates that the rate of CB oxidation over HSiW/CeO_2_ was primarily influenced by the concentrations of surface-adsorbed CB, Ce^4+^ ions, lattice oxygen species, gaseous CB, and surface-adsorbed oxygen species. However, the concentration of gaseous CB was generally not affected by NH_3_. Therefore, the way NH_3_ inhibited the catalytic oxidation of CB over HSiW/CeO_2_ was likely related to the hindrance of CB adsorption, the reduction of the lattice oxygen species, or the reduction of oxygen species adsorbed on the surface.

**Figure 7:**
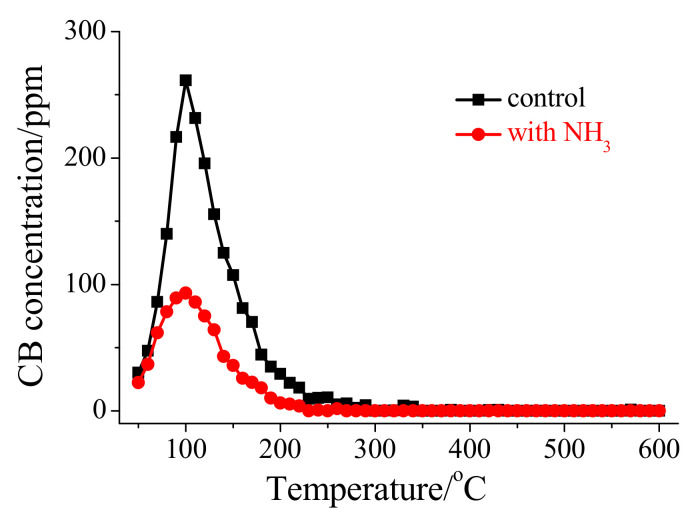
Influence of NH_3_ on CB-TPD profile of HSiW/CeO_2_.

To investigate the impact of NH_3_ on the adsorption of CB onto HSiW/CeO_2_, a CB-TPD analysis was carried out. Figure 7 reveals that the amount of CB adsorbed on HSiW/CeO_2_ was approximately 42.3 μmol g^−1^. However, in the presence of NH_3_, the amount of CB adsorbed on HSiW/CeO_2_ significantly decreased to approximately 18.2 μmol g^−1^ (Figure 7). This only accounted for around 43% of the amount of CB adsorbed in the absence of NH_3_. Therefore, NH_3_ played a dominant role in inhibiting the adsorption of CB onto HSiW/CeO_2_, which may be primarily attributed to the competitive adsorption between NH_3_ and CB molecules.

**Figure 8:**
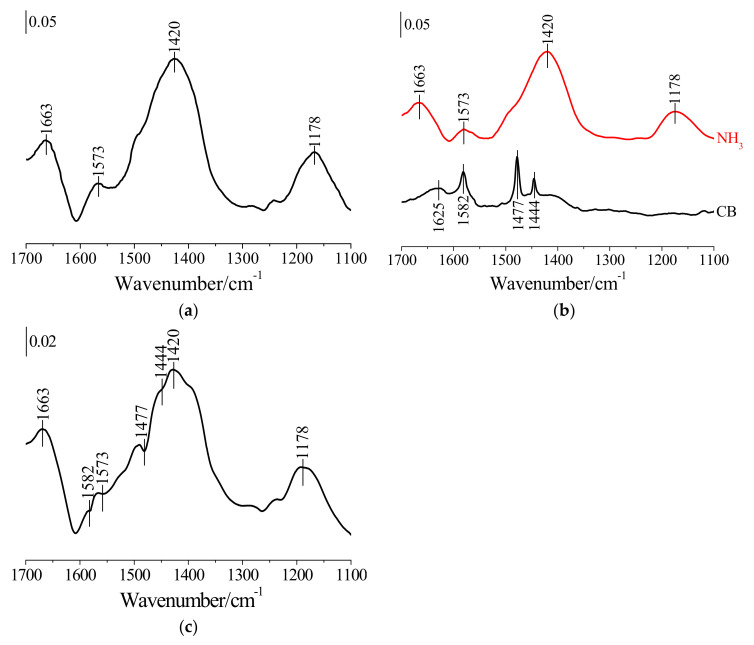
(**a**) In situ DRIFTS spectra of passing NH_3_ over HSiW/CeO_2_ for 30 min at 100 °C. (**b**) In situ DRIFTS spectra of passing NH_3_ over HSiW/CeO_2_ pre-adsorbed by CB for 30 min at 100 °C. (**c**) In situ, DRIFTS spectra resulted from subtracting the spectra of HSiW/CeO_2_ pre-adsorbed by CB after and before NH_3_ adsorption.

To further ascertain the competitive adsorption between NH_3_ and CB onto HSiW/CeO_2_, in situ, DRIFTS of NH_3_ adsorption onto HSiW/CeO_2_ and NH_3_ adsorption onto HSiW/CeO_2_ pre-adsorbed by CB were conducted. After the adsorption of NH_3_ at 100 °C, four distinct bands at 1178, 1420, 1573, and 1663 cm^−1^ appeared on HSiW/CeO_2_ (Figure 8a). The bands at 1178 and 1573 cm^−1^ were attributed to the coordinated NH_3_ adsorbed on Lewis acid sites, which were formed by Ce^3+^/Ce^4+^ in CeO_2_ and W^6+^ in HSiW [17]. On the other hand, the bands at 1420 and 1663 cm^−1^ were ascribed to ionic NH^4+^ adsorbed on Brønsted acid sites, which were formed by Ce–OH in CeO_2_ and W–OH in HSiW [17]. Meanwhile, CB was also adsorbed on Lewis acid sites due to Ce^3+^/Ce^4+^ in CeO_2_ and W^6+^ in HSiW and Brønsted acid sites due to Ce–OH in CeO_2_ and W–OH in HSiW (Figure 4a). These results suggest that NH_3_ and CB generally had common adsorption sites, resulting in the competitive adsorption of NH_3_ and CB onto HSiW/CeO_2_. Furthermore, when NH_3_ was introduced into HSiW/CeO_2_ pre-adsorbed by CB at 100 °C, the bands corresponding to coordinated NH_3_ and ionic NH^4+^ were also observed (Figure 8b). However, it was challenging to differentiate the bands corresponding to adsorbed CB due to the overlapping signals of coordinated NH_3_ and ionic NH^4+^. To address this issue, a subtraction technique was employed by comparing the spectra before and after NH_3_ adsorption. Interestingly, three negative bands at 1444, 1477, and 1582 cm^−1^, which corresponded to CB adsorbed on HSiW/CeO_2_, became significantly apparent (Figure 8c). This suggests that the intensity of bands corresponding to CB adsorbed on HSiW/CeO_2_ decreased substantially following NH_3_ adsorption. Therefore, NH_3_ can displace CB adsorbed on HSiW/CeO_2_, leading to the inhibition of CB adsorption. Moreover, it was possible that NH_3_ could react with HCl produced from CB oxidation over HSiW/CeO_2_, forming NH_4_Cl [33]. This NH_4_Cl formation can cover the surface’s adsorption sites, thereby hindering the adsorption of CB.

**Figure 9:**
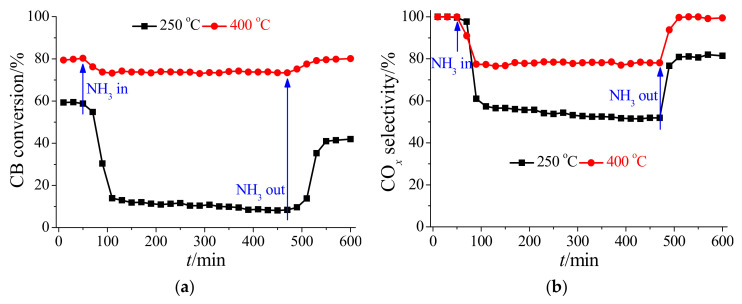
(**a**) CB conversion efficiency and (**b**) CO*_x_* selectivity during the transient reaction of CB oxidation over HSiW/CeO_2_ with NH_3_ at 250 and 400 °C. Reaction conditions: [CB] = 100 ppm, [NH_3_] = 500 ppm (during use), [O_2_] = 5%, catalyst mass = 50 mg, total flow rate = 200 mL min^−1^, and GHSV = 240,000 cm^3^ g^−1^ h^−1^.

To ascertain the formation of NH_4_Cl during CB oxidation over HSiW/CeO_2_, the transient reaction of CB oxidation with NH_3_ was performed at 250 and 400 °C, respectively. After CB oxidation over HSiW/CeO_2_ was stable with approximately 58% conversion efficiency and 100% CO*_x_* selectivity for 50 min at 250 °C, 500 ppm NH_3_ was introduced into the reaction atmosphere (Figure 9). Then, the CB conversion efficiency and CO*_x_* selectivity decreased to approximately 10% and 53%, respectively (Figure 9). This further demonstrated that CB oxidation over HSiW/CeO_2_ was remarkably inhibited by NH_3_. However, the CB conversion efficiency and CO*_x_* selectivity only converted to approximately 41% and 81% when the introduction of NH_3_ into the reaction atmosphere was stopped, respectively (Figure 9). This suggests that NH_4_Cl was formed on HSiW/CeO_2_ during CB oxidation at low reaction temperature, resulting in the deterioration of its performance for CB oxidation. As the reaction temperature increased to 400 °C, the CB conversion efficiency and CO*_x_* selectivity of HSiW/CeO_2_ still decreased after the introduction of NH_3_ into the reaction atmosphere (Figure 9). This also demonstrated that CB oxidation over HSiW/CeO_2_ can be remarkably inhibited by NH_3_. However, the CB conversion efficiency and CO*_x_* selectivity can come back to the original once the introduction of NH_3_ into the reaction atmosphere is stopped (Figure 9). This suggests that little NH_4_Cl was formed on HSiW/CeO_2_ during CB oxidation at high reaction temperatures.

NH_3_ not only competed with CB for the available adsorption sites on HSiW/CeO_2_, but it also had the capability to easily displace already adsorbed CB on the adsorption sites. Moreover, NH_4_Cl formed by the reaction between NH_3_ and HCl covered the same adsorption sites further limiting their availability for CB adsorption. Therefore, the presence of NH_3_ led to a significant inhibition of CB adsorption onto HSiW/CeO_2_.

**Figure 10:**
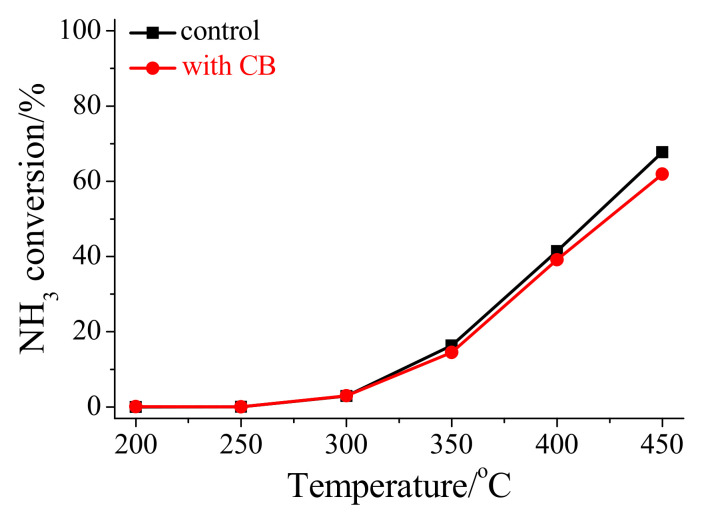
Influence of CB on NH_3_ oxidation over HSiW/CeO_2_. Reaction conditions: [NH_3_] = 500 ppm, [CB] = 100 ppm (during use), [O_2_] = 5%, catalyst mass = 30 mg, total flow rate = 200 mL min^−1^, and GHSV = 400,000 cm^3^ g^−1^ h^−1^.

To further investigate the impact of NH_3_ on the quantities of lattice oxygen species and oxygen species adsorbed on HSiW/CeO_2_, the catalytic oxidation of NH_3_ over HSiW/CeO_2_ was conducted. Figure 10 shows that NH_3_ can be oxidized by HSiW/CeO_2_, with the NH_3_ conversion efficiency of approximately 3–68% at 300–450 °C. Meanwhile, the catalytic oxidation of NH_3_ over HSiW/CeO_2_ was hardly affected by CB (Figure 10). These findings strongly suggest that the presence of NH_3_ significantly reduced the quantities of both lattice oxygen species and oxygen species adsorbed on HSiW/CeO_2_, owing to the oxidation of NH_3_ [34,35]. Moreover, the formation of NH_4_Cl through the reaction between NH_3_ and HCl can lead to the coverage of the surface of HSiW/CeO_2_, thereby resulting in a decrease in the quantities of both lattice oxygen species and adsorbed oxygen species.

NH_3_ not only significantly blocked the adsorption of CB onto HSiW/CeO_2_, but it also noticeably reduced the amount of lattice oxygen species present on HSiW/CeO_2_. Thus, the Mars–van–Krevelen mechanism was greatly hindered by NH_3_. Meanwhile, NH_3_ also greatly reduced the amount of oxygen species adsorbed on HSiW/CeO_2_, leading to significant inhibition of the Eley–Rideal mechanism. In consequence, NH_3_ had a profound inhibitory effect on the catalytic oxidation of CB over HSiW/CeO_2_.

### 3.5. CB Oxidation under a Low GHSV of Normal SCR Condition

**Figure 11:**
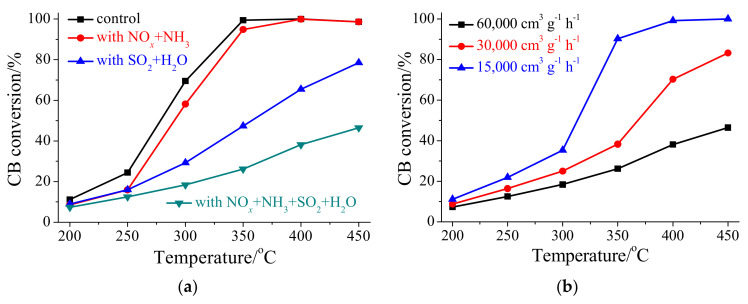
(**a**) CB conversion efficiency of HSiW/CeO_2_ with a low GHSV of normal SCR condition. Reaction conditions: [NO*_x_*] = 500 ppm (during use), [NH_3_] = 500 ppm (during use), [CB] = 100 ppm (during use), [O_2_] = 5%, [SO_2_] = 100 ppm (during use), [H_2_O] = 8% (during use), catalyst mass = 200 mg, total flow rate = 200 mL min^−1^, and GHSV = 60,000 cm^3^ g^−1^ h^−1^. (**b**) Influence of GHSV on CB conversion efficiency of HSiW/CeO_2_. Reaction conditions: [NO*_x_*] = 500 ppm, [NH_3_] = 500 ppm, [CB] = 100 ppm, [O_2_] = 5%, [SO_2_] = 100 ppm, [H_2_O] = 8%, catalyst mass = 200–800 mg, and total flow rate = 200 mL min^−1^.

Figure 11a shows that HSiW/CeO_2_ showed excellent ability for CB oxidation exceeding 350 °C with a CB conversion efficiency of over 90% under a low GHSV of normal SCR conditions. However, the catalytic oxidation of CB over HSiW/CeO_2_ was significantly hindered by NH_3_, and hence HSiW/CeO_2_ hardly functioned as a catalyst for CB oxidation when NH_3_ was present. Nonetheless, as the SCR reaction progressed, the concentration of NH_3_ gradually decreased. Therefore, even though NH_3_ inhibited CB oxidation to a remarkable extent, the surplus HSiW/CeO_2_ catalyst can still drive the catalytic oxidation of CB (Figure S3). This suggests that when NH_3_ is no longer present, CB can still be oxidized by the excess HSiW/CeO_2_ catalyst, resulting in only a slight decrease in CB conversion efficiency upon the introduction of NO*_x_*+NH_3_ (Figure 11a). However, the catalytic oxidation of CB over HSiW/CeO_2_ was severely impeded by SO_2_ and H_2_O, which were inevitable components in flue gas (Figure 11a) [36,37]. Therefore, when NO*_x_*, NH_3_, SO_2_, and H_2_O were all present, HSiW/CeO_2_ exhibited poor ability for CB oxidation, yielding a CB conversion efficiency of less than 47%. However, the CB conversion efficiency of HSiW/CeO_2_ significantly improved with a further decrease in GHSV. In fact, a high CB conversion efficiency (>90%) can still be achieved by HSiW/CeO_2_ in the presence of 500 ppm NO*_x_*, 500 ppm NH_3_, 100 ppm SO_2_, and 8% H_2_O, with a GHSV of 15,000 cm^3^ g^−1^ h^−1^ at temperatures exceeding 350 °C (Figure 11b).

### 3.6. Significance

The catalytic oxidation of CB over HSiW/CeO_2_ was found to be significantly hindered by the presence of NH_3_, suggesting that CB was hardly removed by HSiW/CeO_2_ as a co-benefit of NO*_x_* reduction. Therefore, the simultaneous removal of NO*_x_* and CB over HSiW/CeO_2_ can be regarded as a two-stage process rather than a synergistic one. The first stage of this process involved the reduction of NO*_x_* over HSiW/CeO_2_, and the second stage was the catalytic oxidation of CB over the excess HSiW/CeO_2_ catalyst (Figure S4). It was observed that both the reduction of NO*_x_* and the oxidation of CB over HSiW/CeO_2_ were significantly inhibited by SO_2_ and H_2_O. To maintain efficient conversion efficiencies of NO*_x_* and CB in the presence of SO_2_ and H_2_O, it was necessary to increase the amount of HSiW/CeO_2_ used. However, the specific sequence of NO*_x_* reduction followed by CB oxidation over HSiW/CeO_2_ remained unchanged even in the presence of SO_2_ and H_2_O. Hence, in order to achieve simultaneous removal of NO*_x_* and CB over a single HSiW/CeO_2_ catalyst, it would be more meaningful to focus on improving the individual performances of HSiW/CeO_2_ for NO*_x_* reduction and CB oxidation, respectively.

## 4. Conclusions

The catalytic oxidation of CB over HSiW/CeO_2_ primarily followed two mechanisms, namely the Mars–van–Krevelen mechanism and the Eley–Rideal mechanism. The CB oxidation rate of HSiW/CeO_2_ was determined by several factors, including the concentrations of surface-adsorbed CB, Ce^4+^ ions, lattice oxygen species, gaseous CB, and surface-adsorbed oxygen species. NH_3_ not only significantly blocked the adsorption of CB onto HSiW/CeO_2_, but it also noticeably reduced the amount of lattice oxygen species present on HSiW/CeO_2_. Therefore, the Mars–van–Krevelen mechanism was greatly hindered by NH_3_. Moreover, NH_3_ reduced the amount of surface-adsorbed oxygen species, inhibiting the Eley–Rideal mechanism. As a result, NH_3_ remarkably inhibited the catalytic oxidation of CB over HSiW/CeO_2_. This inhibition of CB oxidation by NH_3_ implies that HSiW/CeO_2_ was not effective in removing CB as a co-benefit of NO*_x_* reduction. Instead, the removal of NO*_x_* and CB over HSiW/CeO_2_ can be considered as two separate processes rather than a synergistic removal. Therefore, it was more meaningful to focus on enhancing the performances of HSiW/CeO_2_ for NO*_x_* reduction and CB oxidation individually in order to achieve simultaneous removal of both pollutants using a single HSiW/CeO_2_ catalyst.

## Figures and Tables

**Table 1:** Reaction kinetic constants of CB oxidation over HSiW/CeO_2_.

	Temperature/°C	/μmol g^−1^ min^−1^		
		*k*_E-R_	*k*_MvK_	R^2^
HSiW/CeO_2_	250	0.008	2.77	0.998
	300	0.021	3.89	0.995
	350	0.060	4.07	0.999
	400	0.132	4.63	0.996
	450	0.170	4.79	0.998

## Data Availability

Data are contained within the article.

## Supplementary Materials

The following supporting information can be downloaded at: https://www.mdpi.com/article/10.3390/ma17040828/s1, Figures S1–S8: N_2_O formation during NO*_x_* reduction over HSiW/CeO_2_, XRD patterns of CeO_2_ and HSiW/CeO_2_, NO*_x_* conversion efficiency of HSiW/CeO_2_ with a high GHSV, two-stage removals of NO*_x_* and CB over HSiW/CeO_2_, NO*_x_* conversion efficiency of HSiW/CeO_2_ with a low GHSV of normal SCR condition, influence of GHSV on NO*_x_* conversion efficiency of HSiW/CeO_2_, Selectivities towards CO_2_, CO, and HCl of HSiW/CeO_2_ and V_2_O_5_-WO_3_/TiO_2_, in situ DRIFTS spectra of passing CB over CeO_2_ and HSiW for 30 min at 100 °C, analysis of chlorinated by-product by GC-MS during CB oxidation over HSiW/CeO_2_ at 400 °C; Table S1: Percentages of Ce and W species on/in HSiW/CeO_2_ and V_2_O_5_-WO_3_/TiO_2_.
