# Supplementary material for: Catalytic Oxidation of Chlorobenzene over HSiW/CeO2 as a Co-Benefit of NOx Reduction: Remarkable Inhibition of Chlorobenzene Oxidation by NH3
Source: Materials (Basel). 2024 Feb 8;17(4):828. doi: 10.3390/ma17040828 (PMC10890138; doi:10.3390/ma17040828)
Supplement: Supplementary file 1 [file materials-17-00828-s001.zip › materials-2822098-supplementary.pdf]

Supporting Information

Catalytic oxidation of chlorobenzene over HSiW/CeO_2 as a co-benefit of NO_x reduction: Remarkable inhibition of chlorobenzene oxidation by NH_3

Leyuan Dong, Keyu Jiang, Qi Shen, Lijuan Xie, Jian Mei*, Shijian Yang

School of Environment & Ecology, Jiangnan University, Wuxi, 214122, P. R. China

Corresponding Author:

* School of Environment & Ecology, Jiangnan University. Telephone: 86-15121199536; E-mail: jsjhmj@126.com (Jian Mei).

Content including: Five pages, one table, and eight figures.

Table S1 Percentages of Ce and W species on/in HSiW/CeO_2 and V_2O_5-WO_3/TiO_2 /%

	Ce	V	W	Ce/W
HSiW/CeO_2 from XPS analysis	21	-	3.7	5.7
HSiW/CeO_2 from XRF analysis	31	-	1.7	18
1% V_2O_5-WO_3/TiO_2 from XRF analysis	-	1.4	9.2	-
3% V_2O_5-WO_3/TiO_2 from XRF analysis	-	5.3	9.1	-

* Corresponding author.

E-mail: jsjhmj@126.com (J. Mei).

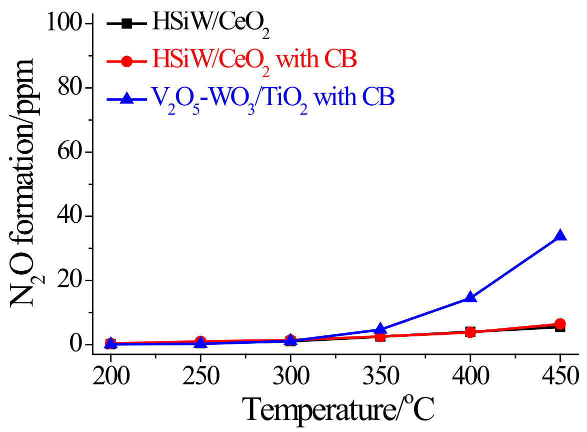

Fig. S1 N_2O formation during NO_x reduction over HSiW/CeO_2. Reaction condition: [NO_x] = 500 ppm (during use), [NH_3] = 500 ppm (during use), [CB] = 100 ppm (during use), [O_2] = 5%, catalyst mass = 30 mg, total flow rate = 200 mL min^{-1}, and GHSV = 400,000 cm^3 g^{-1} h^{-1}.

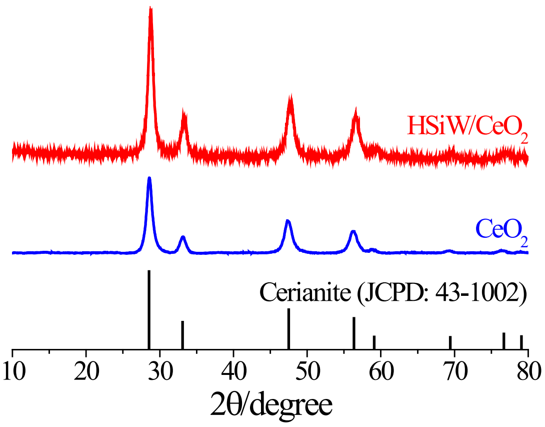

Fig. S2 XRD patterns of CeO_2 and HSiW/CeO_2.

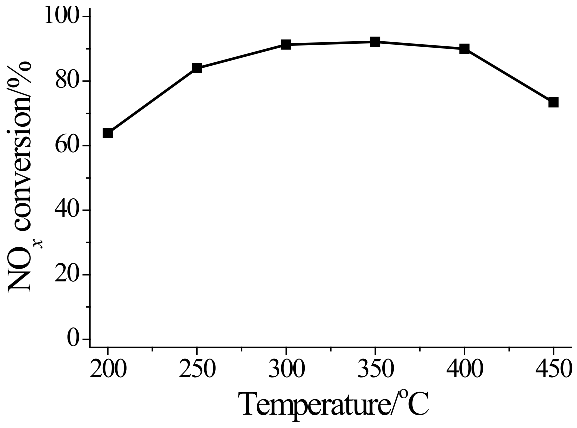

Fig. S3 NO_x conversion efficiency of HSiW/CeO_2 with a high GHSV. Reaction condition: [NO_x] = 500 ppm, [NH_3] = 500 ppm, [O_2] = 5%, catalyst mass = 50 mg, total flow rate = 200 mL min^{-1}, and GHSV = 240,000 cm^3 g^{-1} h^{-1}.

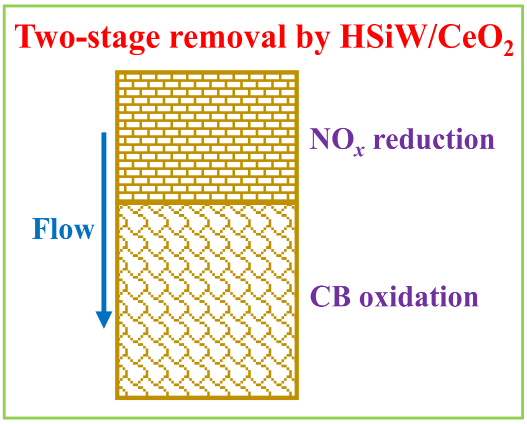

Fig. S4 Two-stage removals of NO_x and CB over $HSiW/CeO_2$.

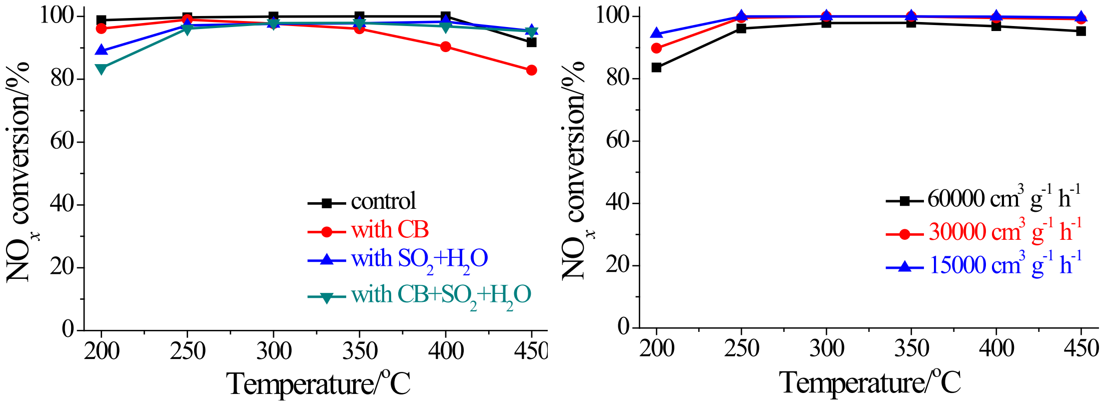

(a) (b)

Fig. S5 (a) NO_x conversion efficiency of $HSiW/CeO_2$ with a low GHSV of normal SCR condition. Reaction condition: $[NO_x]$ = 500 ppm (during use), $[NH_3]$ = 500 ppm (during use), [CB] = 100 ppm (during use), $[O_2]$ = 5%, $[SO_2]$ = 100 ppm (during use), $[H_2O]$ = 8% (during use), catalyst mass = 200 mg, total flow rate = 200 mL min^{-1}, and GHSV = 60,000 cm^3 g^{-1} h^{-1}. (b) Influence of GHSV on NO_x conversion efficiency of $HSiW/CeO_2$. Reaction condition: $[NO_x]$ = 500 ppm, $[NH_3]$ = 500 ppm, [CB] = 100 ppm, $[O_2]$ = 5%, $[SO_2]$ = 100 ppm, $[H_2O]$ = 8%, catalyst mass = 200–800 mg, and total flow rate = 200 mL min^{-1}.

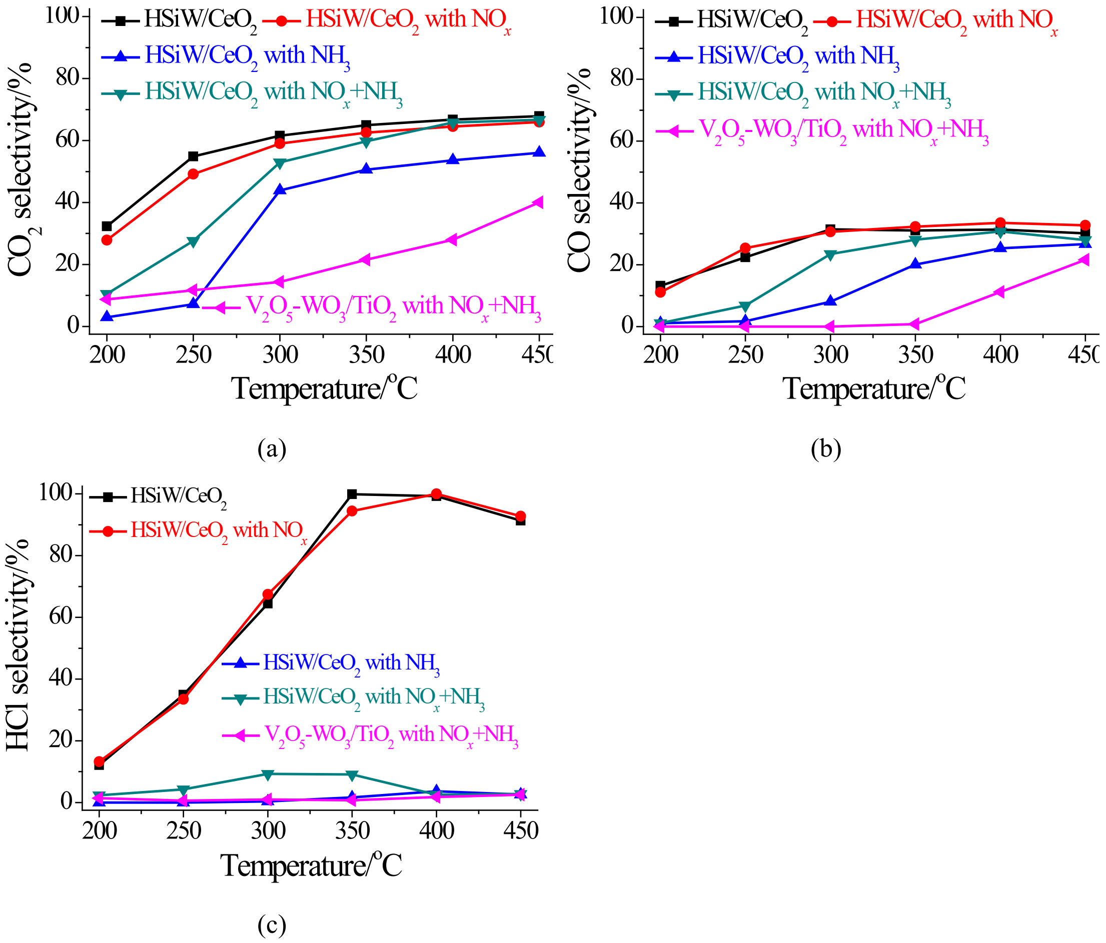

Fig. S6 Selectivities towards (a) CO_2, (b) CO, and (c) HCl of HSiW/CeO_2 and V_2O_5-WO_3/TiO_2. Reaction conditions: [NO_x] = 500 ppm (during use), [NH_3] = 500 ppm (during use), [CB] = 100 ppm (during use), [O_2] = 5%, catalyst mass = 30 mg, total flow rate = 200 mL min^{-1}, and GHSV = 400,000 cm^3 g^{-1} h^{-1}.

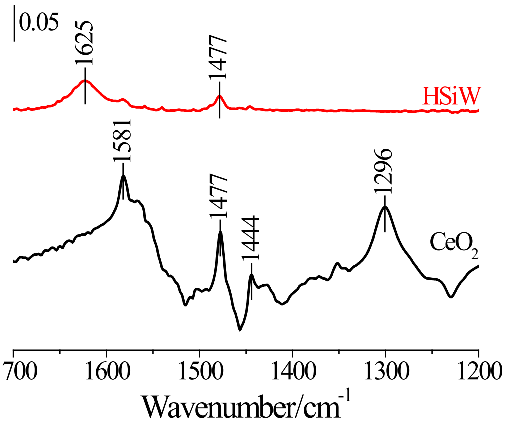

Fig. S7 *In situ* DRIFTS spectra of passing CB over CeO_2 and HSiW for 30 min at 100 °C.

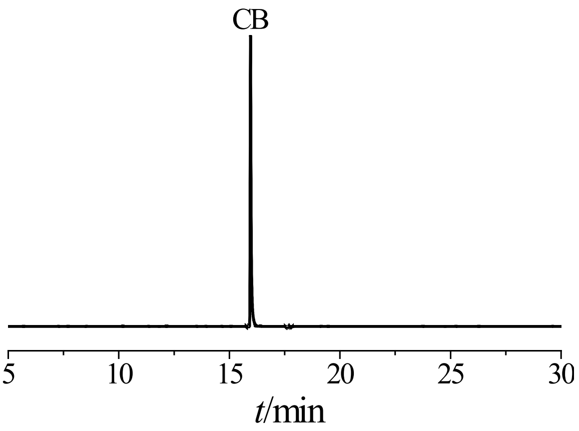

Fig. S8 Analysis of chlorinated by-product by GC-MS during CB oxidation over HSiW/CeO_2 at 400 °C.
